# Tuning the electronic properties and band offset of h-BN/diamond mixed-dimensional heterostructure by biaxial strain

**DOI:** 10.1038/s41598-024-60190-8

**Published:** 2024-04-24

**Authors:** Yipu Qu, Hang Xu, Jiping Hu, Fang Wang, Yuhuai Liu

**Affiliations:** 1 National Center for International Joint Research of Electronic Materials and Systems, International Joint-Laboratory of Electronic Materials and Systems of Henan Province, College of Electrical and Information Engineering, Zhengzhou University, Zhengzhou, 450001 Henan People’s Republic of China; 2 Institute of Intelligence Sensing, Zhengzhou University, Zhengzhou, 450001 Henan People’s Republic of China; 3 Research Institute of Industrial Technology Co. Ltd, Zhengzhou University, Zhengzhou, 450001 Henan People’s Republic of China; 4 Zhengzhou Way Do Electronics Co. Ltd, Zhengzhou, 450001 Henan People’s Republic of China

**Keywords:** h-BN, Diamond, Electronic properties, Band offset, First principles, Condensed-matter physics, Electronic properties and materials, Semiconductors, Surfaces, interfaces and thin films

## Abstract

The h-BN/diamond mix-dimensional heterostructure has broad application prospects in the fields of optoelectronic devices and power electronic devices. In this paper, the electronic properties and band offsets of hexagonal boron nitride (h-BN)/(H, O, F, OH)-diamond (111) heterostructures were studied by first-principles calculations under biaxial strain. The results show that different terminals could significantly affect the interface binding energy and charge transfer of h-BN/diamond heterostructure. All heterostructures exhibited semiconductor properties. The h-BN/(H, F)-diamond systems were indirect bandgap, while h-BN/(O, OH)-diamond systems were direct bandgap. In addition, the four systems all formed type-II heterostructures, among which h-BN/H-diamond had the largest band offset, indicating that the system was more conducive to the separation of electrons and holes. Under biaxial strain the bandgap values of the h-BN/H-diamond system decreased, and the band type occurred direct–indirect transition. The bandgap of h-BN/(O, F, OH)-diamond system increased linearly in whole range, and the band type only transformed under large strain. On the other hand, biaxial strain could significantly change the band offset of h-BN/diamond heterostructure and promote the application of this heterostructure in different fields. Our work provides theoretical guidance for the regulation of the electrical properties of h-BN/diamond heterostructures by biaxial strain.

## Introduction

Two-dimensional/three-dimensional (2D/3D) hybrid heterostructures have attracted extensive interest in exploring molecular-substrate interactions and surface reactivity due to their unique interface structure and electronic properties^1–3^.Hexagonal boron nitride (h-BN) is one of the most attractive 2D materials. It has the characteristics of atomically flat surface without dangling bonds, ultra-wide bandgap (5.96 eV), high thermal conductivity, low thermal expansion coefficient, excellent electrical insulation and corrosion resistance^4^. These properties make h-BN widely used in the fields of electronic, optoelectronic and thermal management devices. H-BN is generally prepared on the catalytically active metal materials (such as Cu, Ni, Co, Pt, etc.), and the research on the interface interaction with the substrate has been widely explored^5,6^. For example, Lin’s team demonstrated that the weak interaction between hexagonal boron nitride nanosheets (h-BNNSs) and Cu (111) substrates allows the transfer of electrons from h-BN/Cu (111) to adsorbed oxygen molecules, leading to its activation^7^. Zhang et al*.* found that the twist angle dependence of the moire pattern in the h-BN/Cu (111) heterostructure can be used to adjust the electronic properties such as bandgap and work function^8^. However, the heterostructure of h-BN thin and catalytic substrates cannot satisfy the needs of various novel device integration. Combining the film with the target substrate by direct growth or transfer, such as GaN^9^, SiO_2_^10^, diamond^11^, etc., is more conducive to exploring the performance potential of more new optical and power electronic devices. Among them, the h-BN/diamond heterostructure interfacial coupling electronic properties have attracted a lot of research.

Diamond is a key material in ultra-wide bandgap (UWBG) semiconductors. Compared with traditional wide bandgap (WBG) semiconductors, diamond has wider bandgap^12^, higher carrier mobility^13^, higher breakdown field strength^14^ and greater thermal conductivity^15^, which makes it show great application prospects in new generation of high power devices and high frequency electronic devices. The mixed-dimensional heterostructure composed of h-BN grown on diamond has unique structural and performance advantages. Sasama et al*.* prepared field effect transistors (FETs) based on h-BN / diamond structure at room temperature, with carrier mobility up to 680 cm^2^·V^−1^·s^−1^^16^. Wang et al*.* pointed out that the carrier behavior and band alignment of h-BN/H-diamond (111) interface can be modulated by twist angle^17^. However, the effect of strain engineering with biaxial strain on the carrier behavior and band offset at the h-BN/diamond (111) interface has not been revealed. In addition, surface transfer doping is also the most effective method to adjust the surface activity of diamond, and various surface treatments have opened up a wider application space of diamond^18^. For example, hydrogen plasma treatment makes diamond exhibit negative electron affinity and promotes the formation of two-dimensional hole gas (2DHG) on the surface of diamond. The p-type characteristics of the surface layer can be used to fabricate low-power Schottky diodes and field-effect transistors (FETs) with good conductivity^19–22^. The surface of diamond shows positron affinity and surface insulation characteristics by surface oxidation^23^. In addition, the fluorinated diamond surface exhibits polar hydrophobicity, and the characteristics of low capacitance current make it an excellent candidate material for electroanalytical applications^24,25^. Therefore, the systematic study of the modulation of biaxial strain on the electronic properties of h-BN and diamond (111) heterostructures with different termination passivation is expected to provide optimization ideas and theoretical guidance for the design of high-performance electronic devices based on h-BN/diamond heterostructures.

In this paper, van der Waals (vdW) heterostructures of h-BN and different terminated diamond (111) (X-diamond, X = H, O, F, OH) were constructed. By changing the lattice constant of the h-BN/X-diamond heterostructure, the regulation from -12% compressive strain to 12% tensile strain is achieved. Based on density functional theory, the stability and interface electronic properties of h-BN and X-diamond heterostructure systems are studied, including interface coupling strength, charge transfer, energy band, density of states and energy band alignment. In addition, the interfacial carrier behavior and band offset of h-BN/X-diamond are regulated by biaxial strain, and the electronic properties of different systems are compared to explore the broader application scenarios of h-BN/diamond heterostructure under biaxial strain.

## Calculation methods

All the calculations are carried out by the plane-wave based PWmat code^26,27^. The generalized gradient approximation perdew-burke-ernzerhof (GGA-PBE)^28^ functional and the optimized norm-conserving vanderbilt pseudopotential^29^ are used for structural optimization, self-consistent calculations, and electronic state analysis. Periodic boundary conditions are applied to all structures in the x and y directions, and a vacuum layer of 15 angstroms is placed in the z direction to avoid interaction between the top and bottom surfaces of the heterostructure. At the same time, the DFT-D3 method is introduced for vdW correction to accurately describe the interaction between molecules^30^. The truncation energy of the plane wave is set to 50 Ryd (~ 680.3 eV), and the energy convergence accuracy and the force of the single atom during the structural relaxation process are set to 1 × 10^–5^ eV·Å^-3^ and 0.02 eV/Å. For the interaction in the Brillouin zone, we use the 4 × 4 × 1 and 7 × 7 × 1 grids in the Monkhorst–Pack special k-point scheme for lattice optimization and self-consistent calculation. The Hyed-Scuseria-Ernzerhof (HSE06) hybrid functional was used to calculate the energy bands of h-BN and diamond primitive cells. The functional has almost no electron self-interaction error and produces accurate electron energy levels. We verified the stability of all structures at 300 K using the NVE method for temperature control and molecular dynamics (MD) simulations by PWmat. The number of simulation steps was set to 500 and the time step was 1 fs. In order to save the computational cost, the PBE functional was used to calculate the energy band, partial density of states (PDOS) and band offset. The optimised structure, charge density difference and contour plots were drawn using Vesta software^31^. The interfacial binding energy (${E}_{BE}$) between the plates is used to describe the stability of the h-BN and X-diamond heterostructures. The expression is as follows :

$${E}_{BE}=\left({E}_{h-BN/X-diamond}-{E}_{X-diamond}-{E}_{h-BN}\right)/{S}_{A}$$

Among them, ${E}_{h-BN/X-diamond},{E}_{X-diamond}$ and ${E}_{h-BN}$ represent the total energy of the heterostructure, the different passivated diamond (111) surface and the original h − BN, respectively. The ${S}_{A}$ represents the cross-sectional area of the unit cell.

## Results and discussions

### Geometric structure and stability of heterostructures

**Figure 1:**
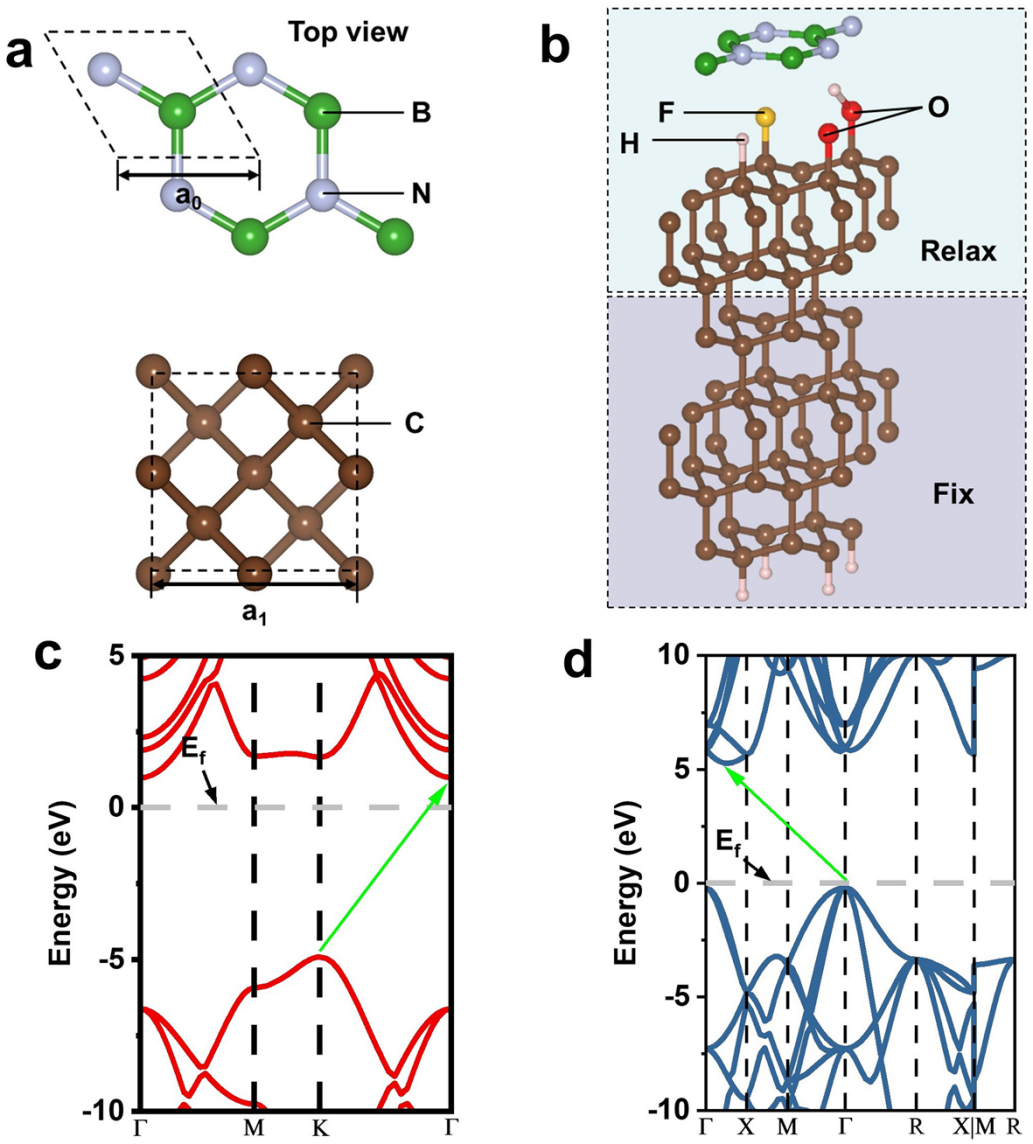
The unit cell structure of (**a**) h-BN and diamond. (**b**) The schematic diagram of h-BN/X-diamond heterostructures. The energy band structures of c. monolayer h-BN and d. bulk diamond calculated by HSE method.

Firstly, the original cells of h-BN and diamond were optimized, as shown in Fig. 1a. The optimized lattice constants of h-BN and diamond were ${a}_{0}$= 2.504 Å^32^ and ${a}_{1}$= 3.535 Å^33^, which were consistent with the conclusions of others. We established the heterostructure of (2 × 2) h-BN(001) / (1 × 1) diamond(111), where the lattice constants of the two parts were 5.008 Å and 5 Å, respectively, with a mismatch of less than 0.2%. Considering that the thickness of the atomic layer is too thin to fully reflect the surface and bulk properties of the material, and taking into account the saving of computing resources, it is necessary to establish an appropriate model thickness for subsequent energy calculations. The layer-projected DOS(LPDOS)^34^ method was used to determine the appropriate diamond (111) atomic layer number of 6 layers (data shown in supplement S1), and the H passivation treatment was performed at the terminal. Figure 1b was a schematic diagram of the heterostructure composed of h-BN and diamond (111) with different terminals (H, O, F, OH). The positions of h-BN, passivation atoms and C atoms at the top two layers of diamond (111) are completely relaxed, and only the bottom atoms are fixed. The bandgaps of monolayer h-BN and bulk diamond were calculated by HSE, and the Fermi level (E_f_) was moved to 0 eV, as shown in Fig. 1c and d. Among them, h-BN and diamond are indirect bandgap semiconductor materials, and their bandgap values are 5.9 eV^35^ and 5.47 eV^36^, respectively, which are close to the experimental values.

**Figure 2:**
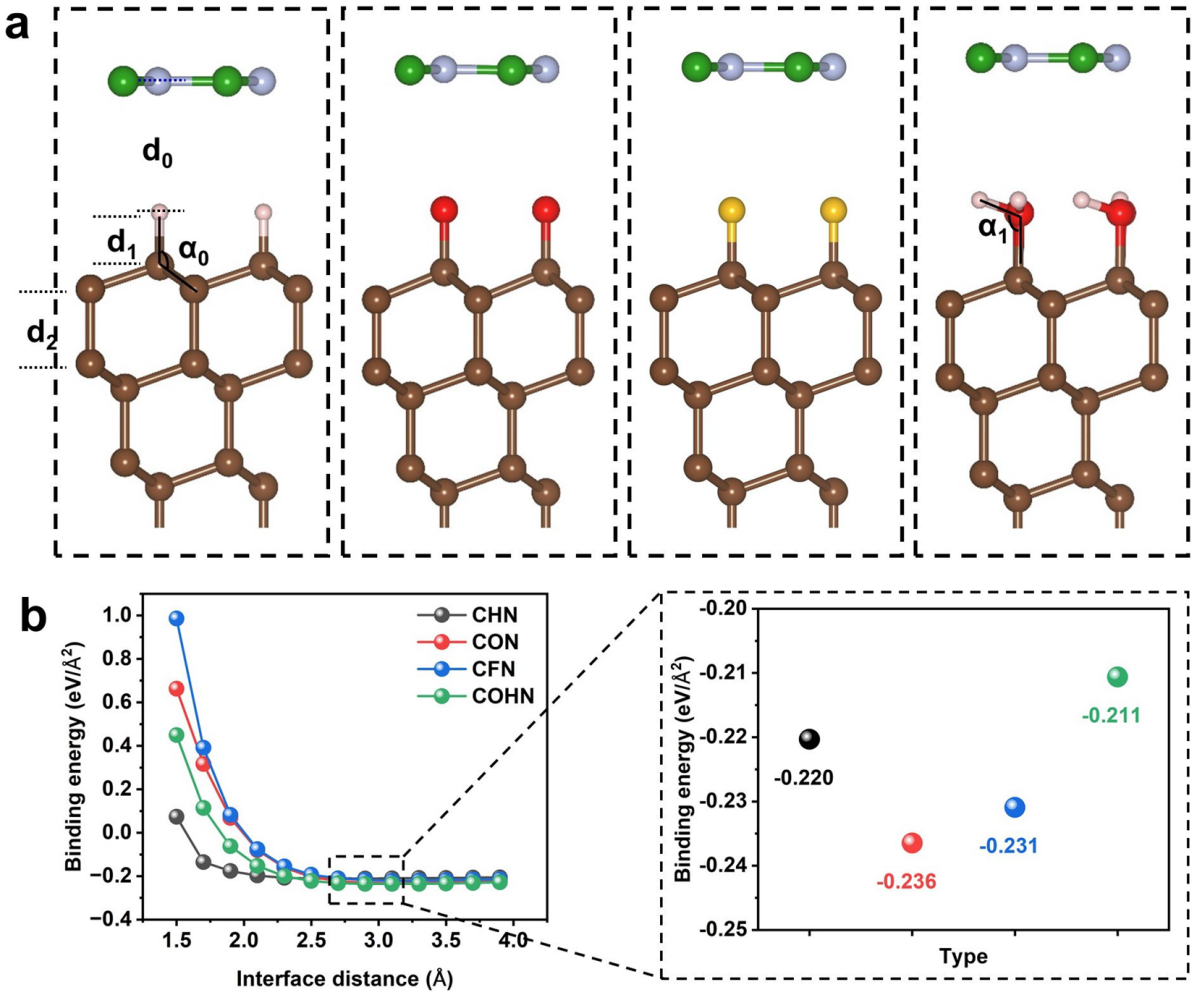
(**a**) Optimized interfacial structure of h-BN/X-diamond. (**b**) The relationship between E_BE_ and interface spacing.

Then, the h-BN / (H, O, F, OH) diamond (111) configuration was fully relaxed to study the interface geometry of the heterostructure. Figure 2a shows the interface atom models of four configurations. In order to obtain the interface model with the most suitable van der Waals spacing, we calculated the relationship between the interface spacing and the E_BE_ of different systems, such as Fig. 2b. The results showed that the most stable interface spacings of the four structures were 2.73, 2.98, 3.05 and 3.02 Å, respectively, and the corresponding E_BE_ were − 0.220, − 0.236, − 0.231 and− 0.236 eV/Å^2^. Obviously, there is no obvious correlation between interface spacing and E_BE_, but all structures can exist. The layer spacing, atomic bond length and bond angle information of each system were sorted out in Table 1. It was found that before and after bonding with h-BN, the bond lengths of C–X in the first layer and C–C in the second layer of the X-diamond system changed less, and the bond length of C–H in H-diamond was the shortest and the bond angle was the smallest. The largest change in bond angle α0 before and after the formation of the heterostructures were for the (H,OH)-terminated diamond system, with an angle change of 0.4°. In the OH-diamond and h-BN/OH-diamond systems, the H–O–C bond angle was only 104.4°, forming a flat surface^37^. In addition, the potential energy fluctuations of all models at room temperature (300 K) were verified by molecular dynamics calculations. The results showed that all systems had good thermal stability (as shown in Fig. S2).

**Table 1:** Interlayer spacing, atomic bond length and bond angle information of X-diamond, h-BN/X-diamond heterostructures.

Heterostructures	Interface layer d_0_ (Å)	Bond length d_1_ (Å)	Bond length d_2_ (Å)	Bond angle α_0_	Bond angle α_1_
H-diamond	–	1.12	1.56	109.1	–
O-diamond	–	1.33	1.53	112.5	–
F-diamond	–	1.38	1.53	109.2	–
OH-diamond	–	1.43	1.54	111.6	104.4
h-BN/H-diamond	2.73	1.1	1.55	108.7°	–
h-BN/O-diamond	2.98	1.32	1.53	112.3°	–
h-BN/F-diamond	3.05	1.37	1.55	109.2°	–
h-BN/OH-diamond	3.02	1.43	1.54	111.2°	104.4°

### Interfacial charge transfer

**Figure 3:**
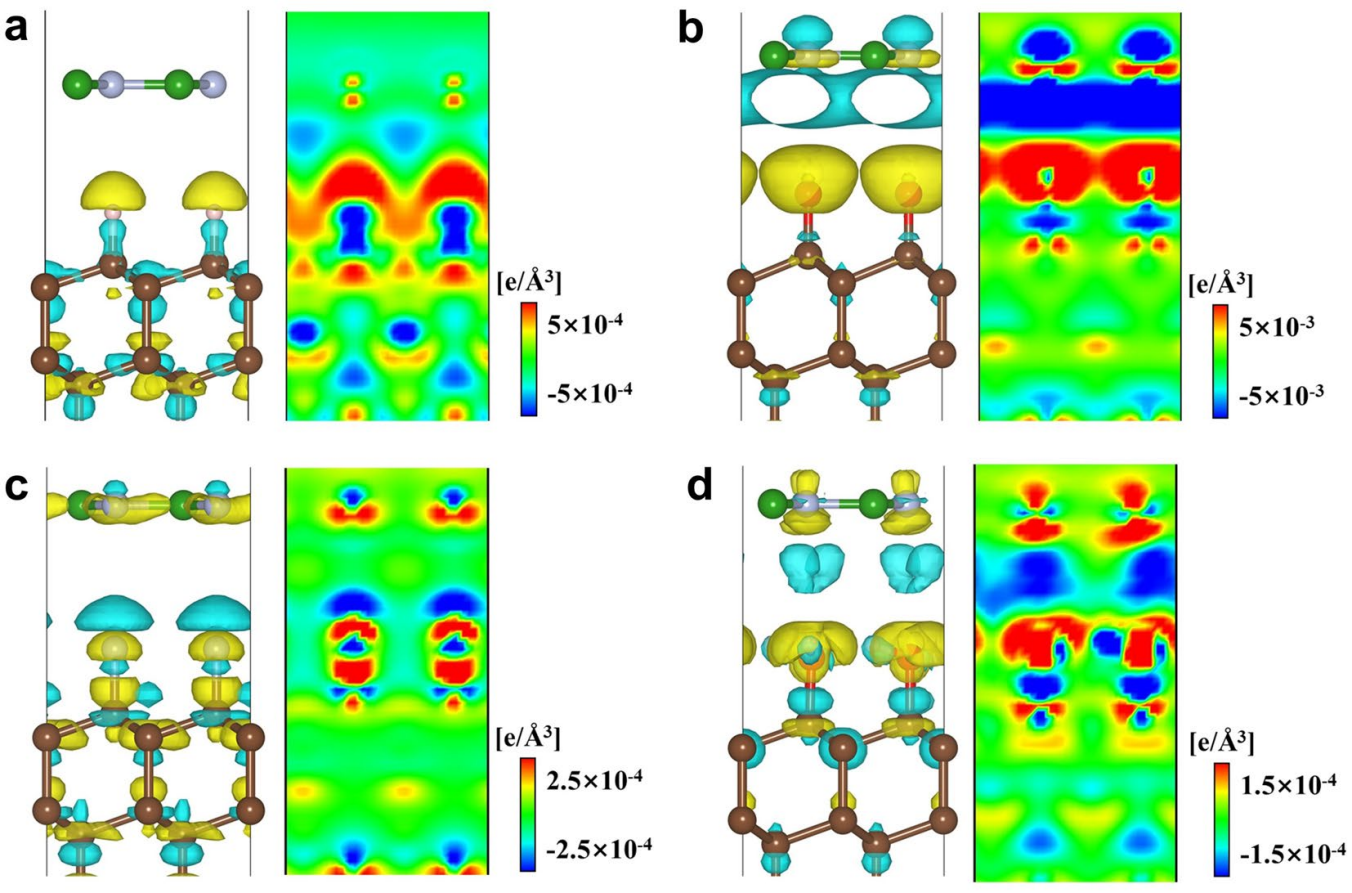
CDD and contour maps of h-BN/X-diamond heterostructures. (**a**) h-BN/H-diamond (**b**) h-BN/O-diamond (**c)** h-BN/F-diamond (**d**) h-BN/OH-diamond. Yellow and cyan represent charge accumulation and depletion, respectively.

In order to clarify the charge transfer at h-BN and (H,O,F,OH) -diamond interface, we use the formula $\Delta\uprho ={\rho }_{h-BN/X-diamond}-{\rho }_{h-BN}-{\rho }_{X-diamond}$ to calculate the charge density difference (CDD) of the system. Here, ${\rho }_{h-BN/X-diamond}$,${\rho }_{X-diamond}$ and ${\rho }_{h-BN}$ represent the charge density of heterostructure, X-diamond surface and h-BN, respectively. Figure 3 was the contour map of CDD and along the (100) plane. The Hirshfeld algorithm was used to decompose the charge, and the output of each part of the charge Q was shown in Table 2. ${\mathbf{Q}}_{{\varvec{B}}{\varvec{N}}},{\mathbf{Q}}_{{\varvec{X}}},{\mathbf{Q}}_{{\varvec{c}}1}$ and ${\mathbf{Q}}_{{\varvec{c}}2}$ represent the charge of BN, terminal atoms, the first and second layer of C atoms on the diamond surface. It could be seen from Fig. 3a that the charge transferred in the h-BN/H-diamond system was mainly concentrated near the C-H bond, and the charge redistribution produces positive and negative dipoles, forming a related potential drop perpendicular to the diamond surface. Therefore, a highly conductive two-dimensional hole gas (2DHG) was formed on the C surface, indicating that hydrogen termination played a key role in the formation of conductivity on the diamond surface^38^. In the h-BN/O-diamond system (Fig. 3b), more charge transfer occured at the h-BN and O-diamond interfaces, indicating that the coupling strength of the interface was large, which was consistent with the E_BE_. Because the electronegativity of O was greater than C, a large number of electrons were gathered around the O atom, forming an inverted surface dipole C^δ+^–O^δ-^^18^. Compared with the h-BN/H-diamond system, the charge distribution of the C–F bond was completely different from that of the C–H bond. The charge of the C–F bond was aggregated, and more charge were transferred to the surface of the F, resulting in the depletion of the surface charge of the diamond (as shown in Fig. 3c). The electric field of the highly polar fully F surface decayed extremely fast, which also resulted in a weak bonding between the h-BN and O-diamond interfaces^24^. At the same time, the correlation between the interface coupling strength and the interface charge transfer can be established by Table 2.

**Table 2:** Electricity Q of different layers of h-BN/(H, O, F, OH)-diamond heterostructures (Unit: e/Å^3^).

Heterostructure	${{\text{Q}}}_{BN}$	${{\text{Q}}}_{X}$	${{\text{Q}}}_{C1}$	${{\text{Q}}}_{C2}$
h-BN/H-diamond	− 0.020	− 0.123	0.130	− 0.002
h − BN/O-diamond	− 0.130	0.327	− 0.192	0.003
h-BN/F-diamond	0.054	0.150	− 0.222	0.002
h-BN/OH-diamond	0.002	0.193	− 0.185	− 0.002

### Projected energy band and density of states

**Figure 4:**
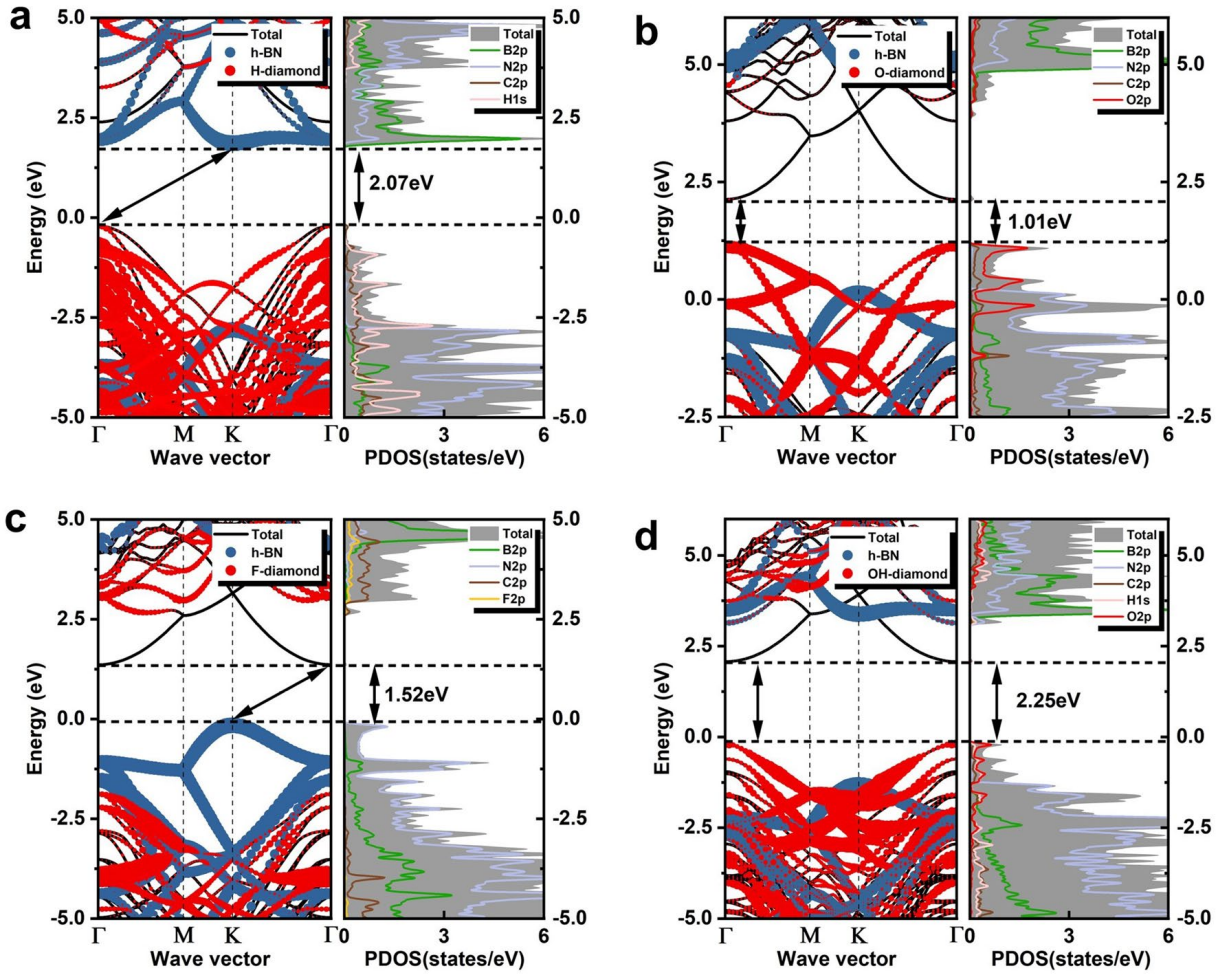
The projected energy band and the corresponding PDOS. (**a**) h-BN/H-diamond (**b**) h-BN/O-diamond (**c**) h-BN/F-diamond (**d)** h-BN/OH-diamond.

The projected energy bands and corresponding partial density of states (PDOS) of the four heterostructures were further calculated. Although COHP (Crystal Orbital Hamilton Population)^39^ has a more precise interpretation of orbital bonding, the high computational cost makes us choose it carefully. In Fig. 4, all heterostructures showed typical semiconductor properties, and the Fermi levels were shifted to 0 eV. As shown in the figure, the bandgaps of the heterostructures composed of h-BN and H-, O-, F-, and OH-terminated diamond (111) surfaces were 2.07, 1.01, 1.52, and 2.25 eV, respectively, indicating that the different terminals affected the interface states of diamond and h-BN. It could be seen that the H and F terminals led to direct to indirect bandgap transitions of the heterostructure, while the O and OH terminals were still the direct bandgap. Figure 4a showed that the minimum conduction band (CBM) of the h-BN/H-diamond system depended on the contribution of h-BN, and the maximum valence band (VBM) was provided by H-diamond. In contrast, CBM and VBM of the h-BN/F-diamond system were at the K point and Γ point in the Brillouin zone in Fig. 4c. In addition, the bandgap of interfacial diamond atoms in the h-BN/H-diamond system was 2.15 eV, which was 61% smaller than the bandgap corresponding to the bulk. The bandgap reduction can be attributed to the underestimation of the bandgap by the PBE function and surface transfer doping. Compared with the O-, F- and OH-terminated models, the VBM of H-terminated diamond was higher than the unoccupied electron energy level of h-BN, so the electrons in the valence band were transferred to the h-BN acceptor and formed ionized holes on the H-diamond surface. By analyzing the PDOS of the h-BN/X-diamond heterostructure, we can further understand the role of h-BN and terminated atoms in regulating electrical properties. In Fig. 4a, the B2*p* and N2*p* orbitals formed CBM, and the hybrid states of C2*p* and H1*s* contributed to the VBM near the Fermi level. In Fig. 4b, c and d, it is clear that for the h-BN/(O,F,OH)-diamond three models, the orbitals of the C atoms are the main contribution to the CBM. In addition for the h-BN/(O,OH)-diamond system, O2*s* and O2*p*z also contribute to the CBM (shown in supplementary materials Fig. S3). For the h-BN/(O,OH)-diamond system, C2*p* and O2*p* contribute primarily to the VBM, and H1s contribute secondarily to the VBM in the h-BN/OH-diamond system. However, the VBM in the h-BN/F-diamond system is almost entirely contributed by the N2*p* orbitals.

### Band offset of heterostructure

**Figure 5:**
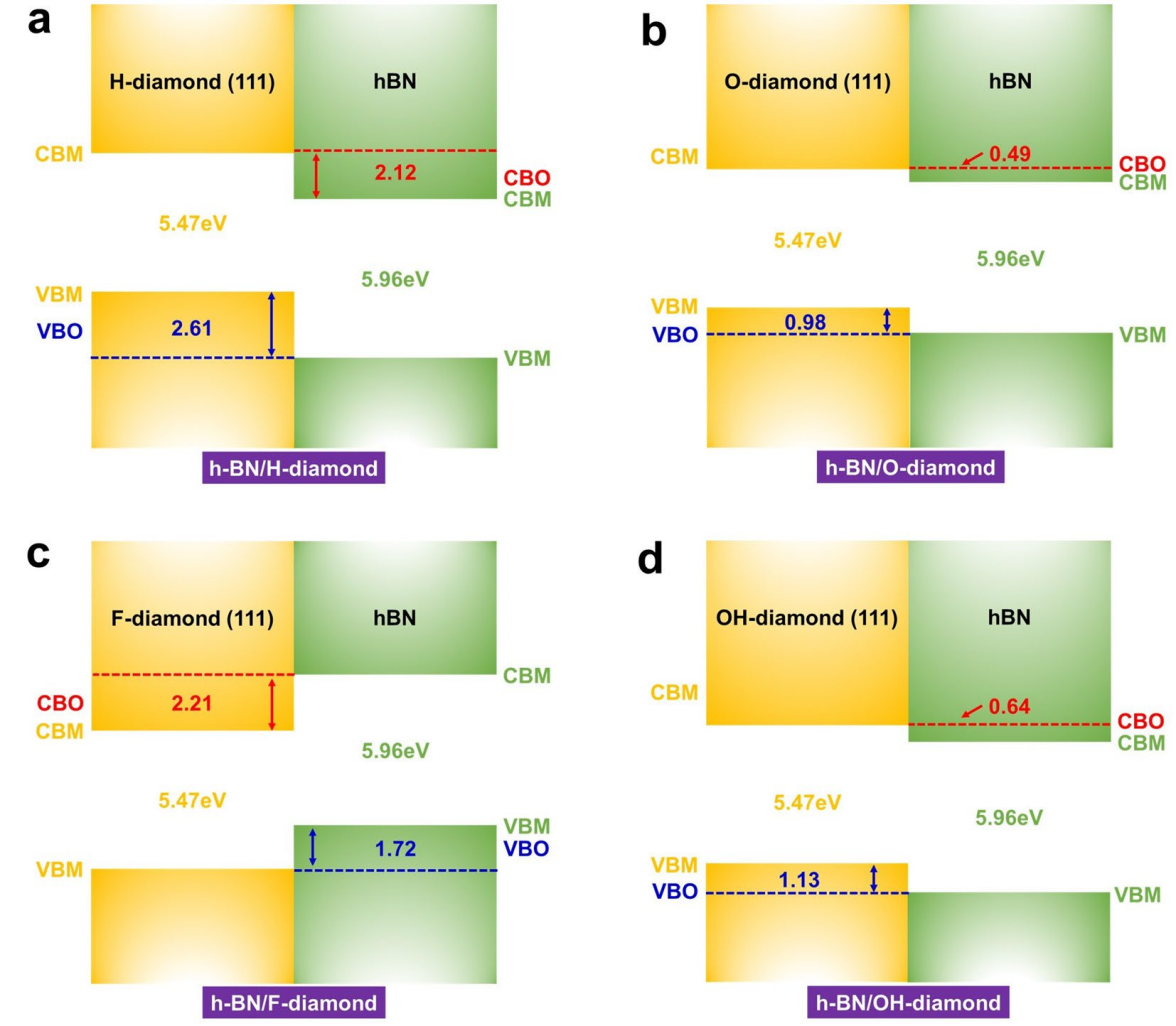
The band offset of heterostructures. (**a**) h-BN/H-diamond (**b**) h-BN/O-diamond (**c**) h-BN/F-diamond (**d**) h-BN/OH-diamond.

In order to analyze the influence of terminated atoms on the electrical properties of h-BN/diamond heterostructure, we further studied the band offset of four systems by using the method of local density of states (LDOS)^34^, as shown in Fig. 5. The calculation formula of conduction band offset (CBO) and valence band offset (VBO) were as follows: CBO = Eg1-Eg2-VBO. In this work, Eg1 and Eg2 were set as the experimental bandgaps of diamond and h-BN, respectively, 5.47 and 5.96 eV. The band offset of the heterostructure caused by different terminals is significant. The H-BN and (H, O, F, OH)-terminated diamond heterostructure established typical type II band offset. It could be seen that their VBOs were 2.61, 0.98, 1.72 and 1.13 eV, respectively. The VBO of the H-terminated system was close to the work of others^17^, indicating the reliability of our results. Compared with other systems, the h-BN/H-diamond heterostructure had a larger VBO, indicating a stronger hole confinement. During the photoexcitation process, electrons will transfer from the local state of diamond to the local state of h-BN and accumulate on the side of h-BN, while holes will accumulate on the surface of H-diamond to form a surface conductive layer. Among them, h-BN is used as an electron acceptor and diamond is used as an electron donor. This system promoted the spontaneous separation of electrons and holes and reduces the risk of leakage current generation. Obviously, this process was opposite in the h-BN/F-diamond system. Compared with the above two systems, the h-BN/OH-diamond system had a weaker electron binding ability, and the CBO was only 0.64 eV. In addition, the VBO of the h-BN/O-diamond heterostructure was less than 1 eV and prone to leakage risk and required additional insulators to ensure the stability of the device^40^.

### Band structure under biaxial strain

**Figure 6:**
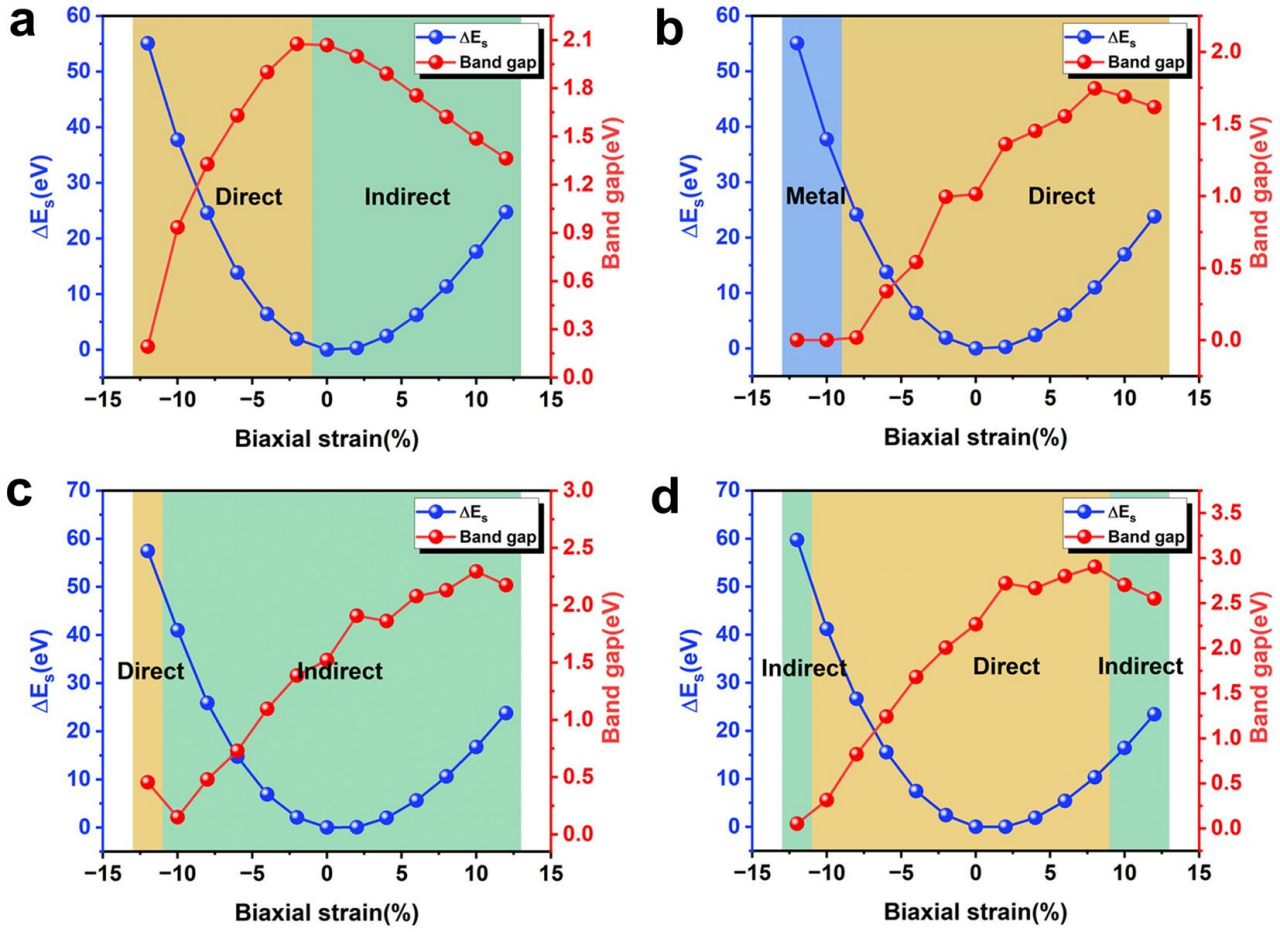
Transformation in strain energy and bandgap of different systems under biaxial strain of − 12–12%. (**a**) h-BN/H-diamond (**b**) h-BN/O-diamond (**c**) h-BN/F-diamond d. h-BN/OH-diamond.

The dissimilarity of the symmetric surface structure between the 2D material and the substrate will undoubtedly cause the in-plane biaxial strain, which has a crucial influence on the structure of the 2D/3D heterostructure, the van der Waals coupling strength and the electronic properties at the interface. Biaxial strain is an effective method to regulate the photoelectric properties of mixed-dimensional heterostructures, which is helpful to expand the wide application of h-BN/diamond heterostructure in the fields of nanoelectronics and optoelectronics. In this section, we discuss the corresponding change of strain energy and bandgap when applying biaxial strain from − 12 to 12% to the h-BN/X-diamond heterostructure. Here the strain (ε) was defined as^41^:

$$\varepsilon =\left(L-{L}_{0}\right)/L$$

where $L$ and ${L}_{0}$ were the lattice constants of h-BN/X-diamond heterostructure with and without strain, respectively. ε > 0 indicated tensile strain, and ε < 0 indicated compressive strain. In addition, the strain energy ($\Delta {E}_{s}$) was defined as :

$$\Delta {E}_{s}={E}_{strained}-{E}_{unstrained}$$

where ${E}_{strained}$ and ${E}_{unstrained}$ were the system total energy of h-BN/X-diamond heterostructure with and without strain, respectively. Figure 6 showed the changes of strain energy and bandgap of different systems under biaxial strain of − 12–12%, and the energy band diagrams of partial strain were placed in the supplement Fig. S4. It can be seen from Fig. 6 that all systems were elastically responsive to strain, and their strain energy curves were smooth parabolas. Whether ε > 0 or ε < 0, the strain energy increased linearly. And only when ε = 0, the strain energy was the smallest. In addition, the adjustment of the energy band of the system under biaxial strain was obvious. In Fig. 6a, the bandgap of h-BN/H-diamond decreased linearly regardless of compressive strain or tensile strain. In the range of − 12% < ε < − 2%, the system exhibited direct bandgaps, and in the range of 2% < ε < 12%, the system showed indirect bandgaps. The energy competition near the band edge caused the bandgap to undergo a direct–indirect transition, with the CBM changing from Γ to K and the VBM staying at Γ. The different bandgap evolutions under biaxial strain are closely related to the bonding nature of the orbitals (as shown in supplementary materials Fig. S5a). The energy band change trend of h-BN/(O, F, OH)-diamond under biaxial strain were completely different from that of H-terminated system. Under the strain from − 12 to 12%, the energy bands of these three systems showed approximately linear increase trend. As shown in Fig. 6b, the large compressive strain of − 10 and − 12% caused the bandgap of h-BN/O-diamond to transform from direct bandgap to zero bandgap, and the system exhibited metal characteristics. It is shown that the heterostructure is mechanically tough and has controlled electrical conductivity and transmittance. Figure 6c showed that the bandgap of h-BN/F-diamond transformed from indirect bandgap to direct bandgap only when the compressive strain of − 12% was applied. However, the bandgap type of h-BN/OH-diamond didn’t not change under the strain of − 10% < ε < 10%. Only when ε = − 12% or ε ≥ 10% the bandgap transformed from direct bandgap to indirect bandgap, which was due to a change in the contributing orbitals of the CBM (as shown in supplementary materials Fig. S5b). The above results showed that appropriate biaxial strain can effectively modulate the bandgap width and type of h-BN/(H, O, F, OH)-diamond, and induce indirect-direct bandgap transition and semiconductor–metal transition, which was expected to achieve potential applications in power electronic devices and optoelectronic devices.

### Band offset under biaxial strain

**Figure 7:**
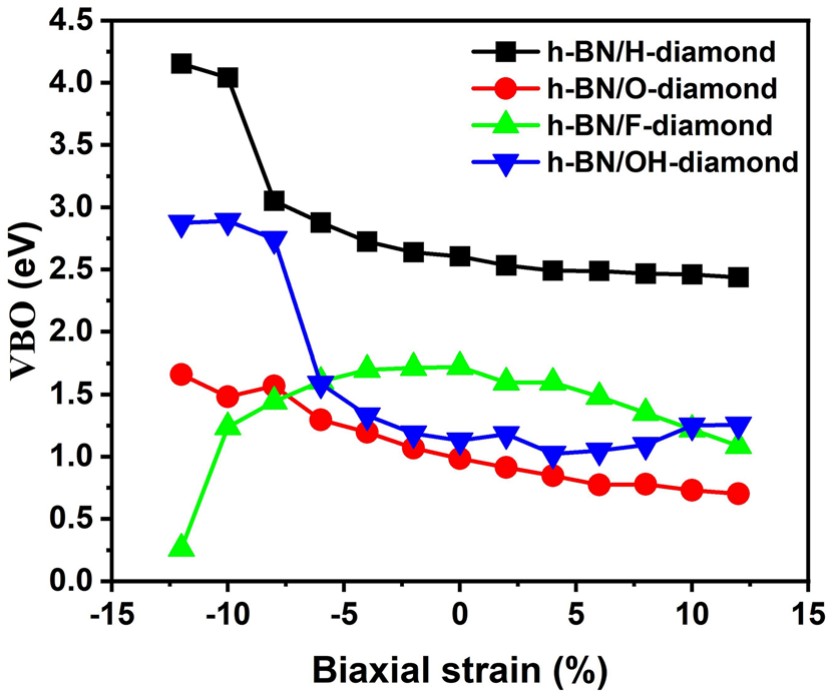
Effect of biaxial strain on band offset of different systems.

Furthermore, we investigated the effect of biaxial strain on VBO, as shown in Fig. 7. Biaxial strain changes the atomic spacing in the crystal cell, which affects the distribution of overlapping atomic orbitals^42^. At the same time, the charge transfer between the diamond and h-BN monolayers is also affected by biaxial strain (as shown in supplementary material Fig. S6), which modifies the internal electric field formed in the heterostructure. Under the same strain, the VBO of h-BN/H-diamond heterostructure was always larger than that of other systems. On the whole, the VBO of h-BN/(H, O, OH)-diamond showed downward trend in the range of − 12% < ε < 12%, while the VBO of h-BN/F-diamond increased firstly and then decreased. As we can see from the figures, the VBOs of h-BN/(H, OH)-diamond system were greater than 1 eV in the full strain range, and the h-BN/F-diamond only was greater than 1 eV in the range of − 10% < ε < 12%. The VBO of h-BN/O-diamond was greater than 1 eV in the range of − 12% < ε < 0, and the VBO was less than 1 eV in the range of ε ≥ 0, indicating that the system may promote the efficient recombination of electron–hole pairs in space under tensile strain, which was helpful to realize the application of h-BN/diamond heterostructure in the field of solar cells and improve its luminous efficiency.

## Conclusion

In summary, the effects of biaxial strain on the electronic structure and band offset between monolayer h-BN and (H, O, F, OH)-terminated diamond (111) were studied by DFT-based first principles method. The results showed that the surface termination can regulate the coupling strength between h-BN and diamond surface. From the interface binding energy, the interface of h-BN/O-diamond was the most stable, and the results were consistent with the charge transfer. The effect of biaxial strain on bandgap and band offset is obvious. Without strain, all heterostructures exhibited semiconductor properties, where the h-BN/(H, F)-diamond system was indirect bandgap and the h-BN/(O, OH)-diamond systems were direct bandgap. This suggests an important contribution of biaxial strain to the bandgap modulation, with direct-gap to indirect-gap and semiconductor-to-metal transitions occurring in the elastic response range. The experimental integration of h-BN/diamond heterostructure holds great promise for the future in nanoelectronics due to good thermal stability and appropriate lattice mismatch. In addition, the VBO and CBO of H/F-terminated diamond were both greater than 1 eV, indicating that the electron–hole pairs of these two systems could be effectively constrained and the leakage current of the related device could be reduced. When ε < 0 or ε > 0, the bandgap of the h-BN/H-diamond system was reduced, and the types of bandgap were different. When − 12% < ε < 12%, the bandgap of h-BN/(O, F, OH)-diamond systems increased linearly, and the bandgap type only transformed under large strain. In terms of band offset, whether it was compressive strain or tensile strain, h-BN/(H, F, OH)-diamond heterostructure had great application potential in the field of photodetectors and photocatalysis, and h-BN/O-diamond heterostructure under tensile strain could be used as alternative material for efficient solar cells. Our work proved that biaxial strain was an effective method to adjust the electrical properties of h-BN/diamond systems, which broadens the application of h-BN/diamond hybrid dimensional heterostructure in optoelectronic devices and power electronic devices.

### Supplementary Information

Supplementary Information.

## Data Availability

The code of theoretical calculation and the graphic data for analysis in this paper can be obtained from the corresponding author Yipu Qu according to reasonable requirements.
