# Supplementary material for: Tuning the electronic properties and band offset of h-BN/diamond mixed-dimensional heterostructure by biaxial strain
Source: Sci Rep. 2024 Apr 24;14:9414. doi: 10.1038/s41598-024-60190-8 (PMC11043405; doi:10.1038/s41598-024-60190-8)
Supplement: Supplementary file 1 — Supplementary Information. [file 41598_2024_60190_MOESM1_ESM.docx]

# Supporting Information

# Tuning the electronic properties and band offset of h-BN/diamond mixed-dimensional heterostructures by biaxial strain

Yipu Qu*^1^**,** Hang Xu^1^, Jiping Hu^1^, Fang Wang*^1, 2, 3, 4^, Yuhuai Liu*^1, 2, 3, 4^

1. National Center for International Joint Research of Electronic Materials and Systems, International Joint-Laboratory of Electronic Materials and Systems of Henan Province, college of Electrical and Information Engineering, Zhengzhou University, Zhengzhou, Henan 450001, P. R. China

2. Institute of Intelligence Sensing, Zhengzhou University, Zhengzhou, Henan 450001, P. R. China

3. Research Institute of Industrial Technology Co. Ltd., Zhengzhou University, Zhengzhou, Henan 450001, P. R. China

4. Zhengzhou Way Do Electronics Co. Ltd., Zhengzhou, Henan 450001, P. R. China

E-mails: [ieypqu@zzu.edu.cn](mailto:ieypqu@zzu.edu.cn); [iefwang@zzu.edu.cn](mailto:iefwang@zzu.edu.cn); [ieyhliu@zzu.edu.cn](mailto:ieyhliu@zzu.edu.cn)


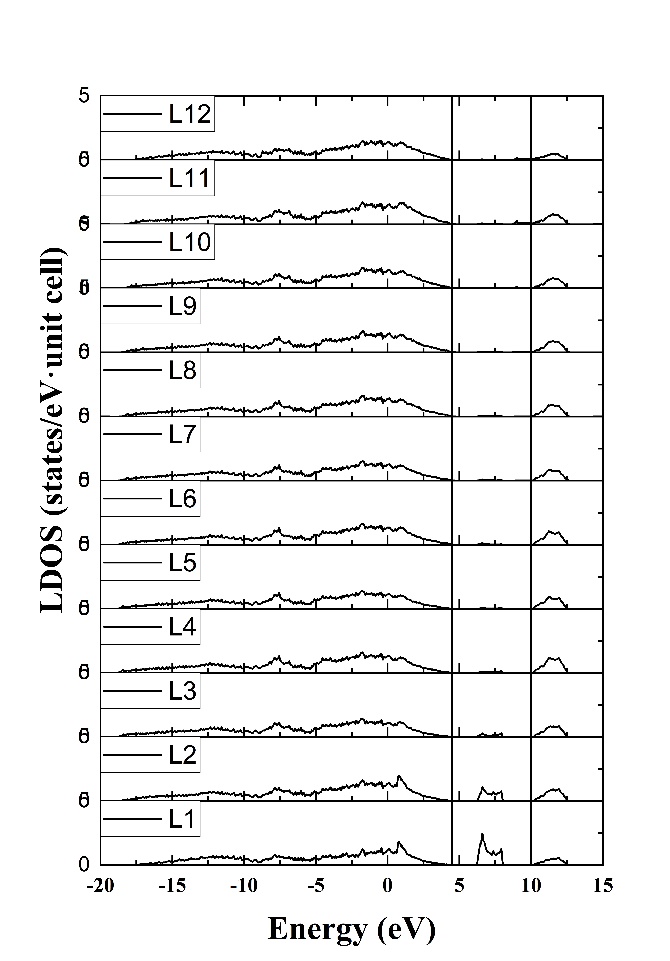


**Figure S1. The LDOS of diamond (111) surface.**


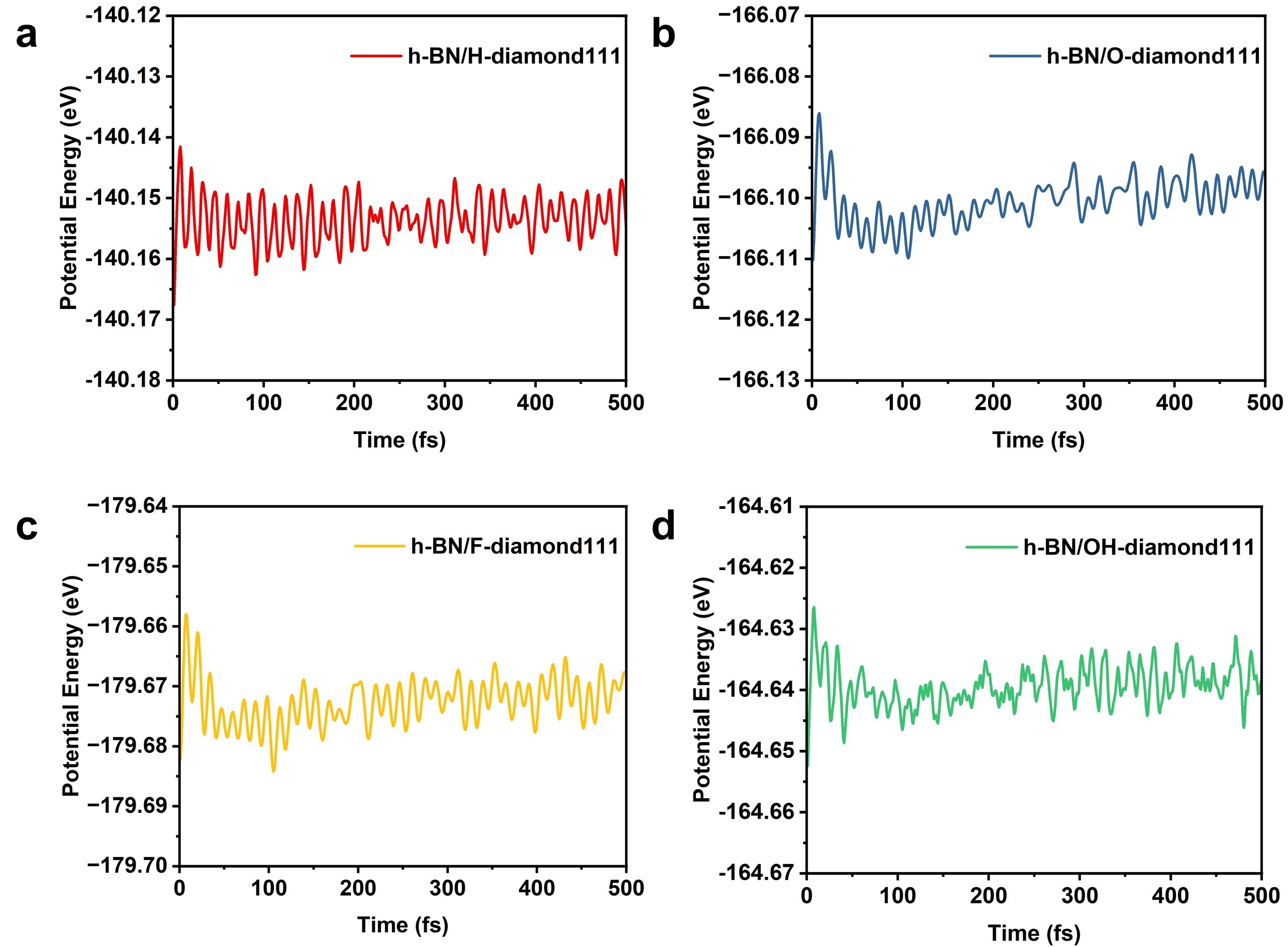


**Figure S2. The potential energy of a. h-BN/H-diamond b. h-BN/O-diamond c. h-BN/F-diamond d. h-BN/OH-diamond at room temperature (300K).**


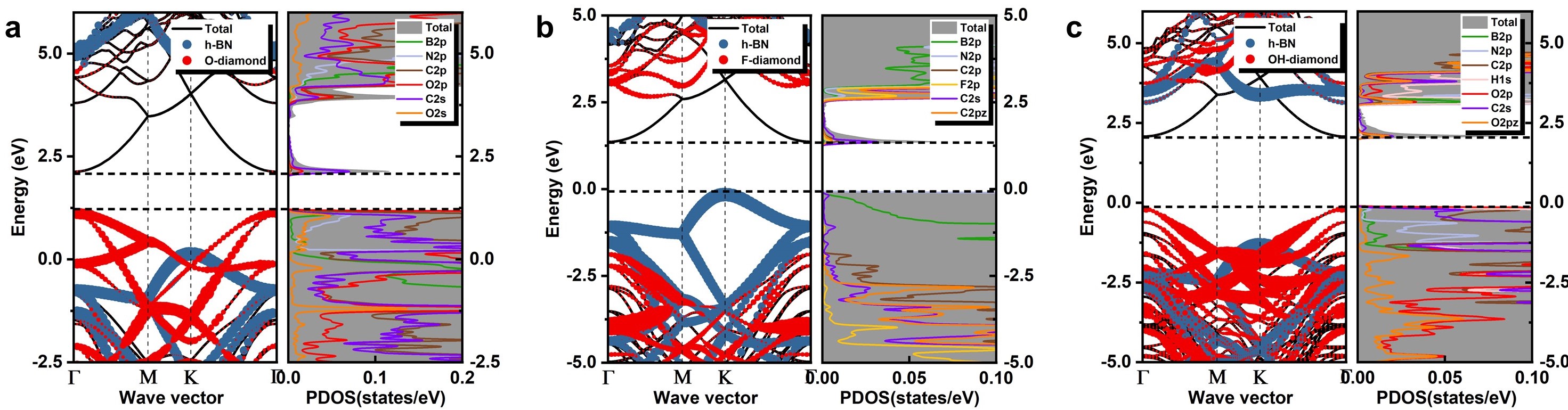


Figure S3. Band structure and PDOS of h-BN/(O,F,OH)-diamond.


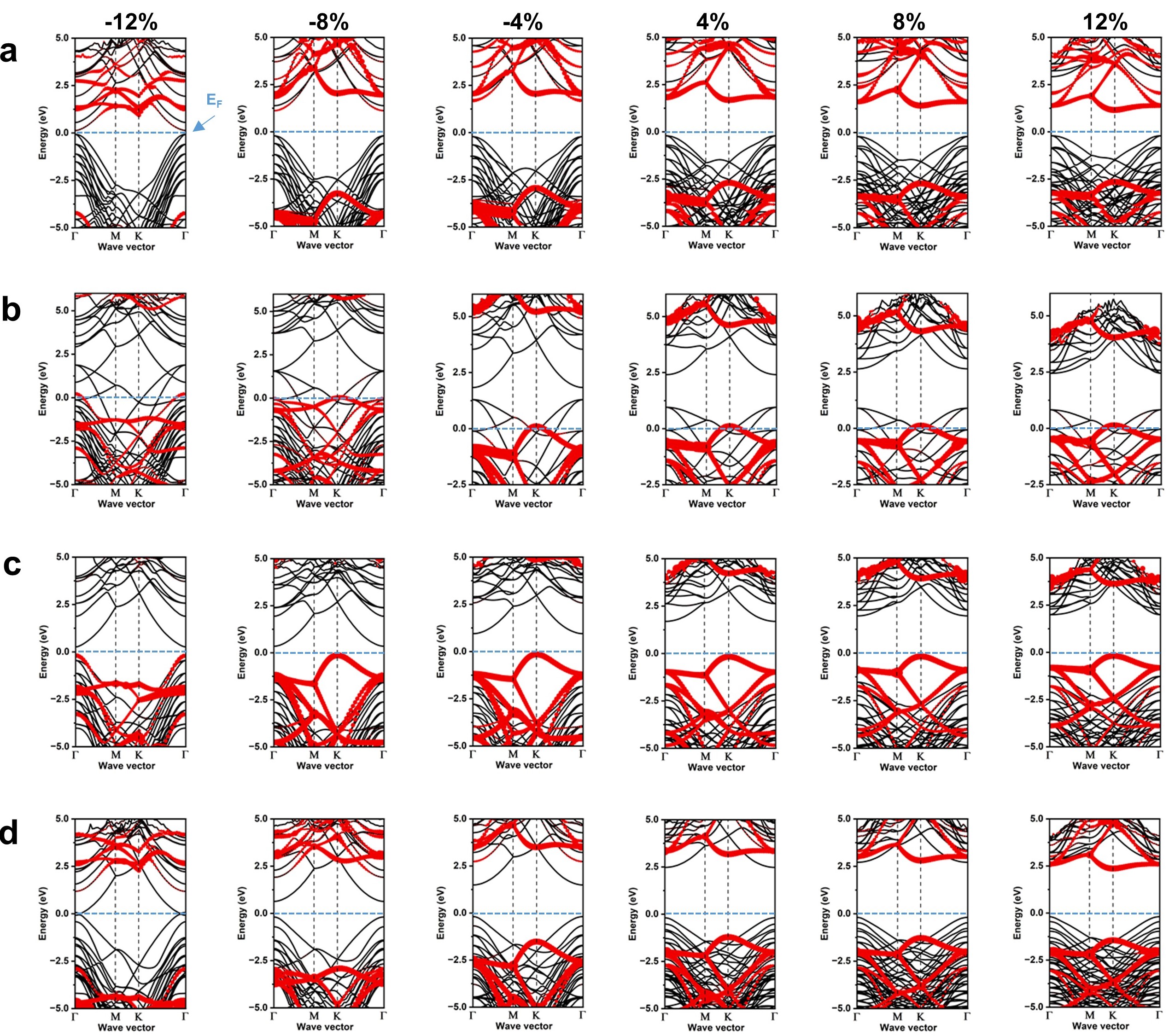


**Figure S4. The bandstructure of a. h-BN/H-diamond b. h-BN/O-diamond c. h-BN/F-diamond d. h-BN/OH-diamond under partial biaxial strain.**


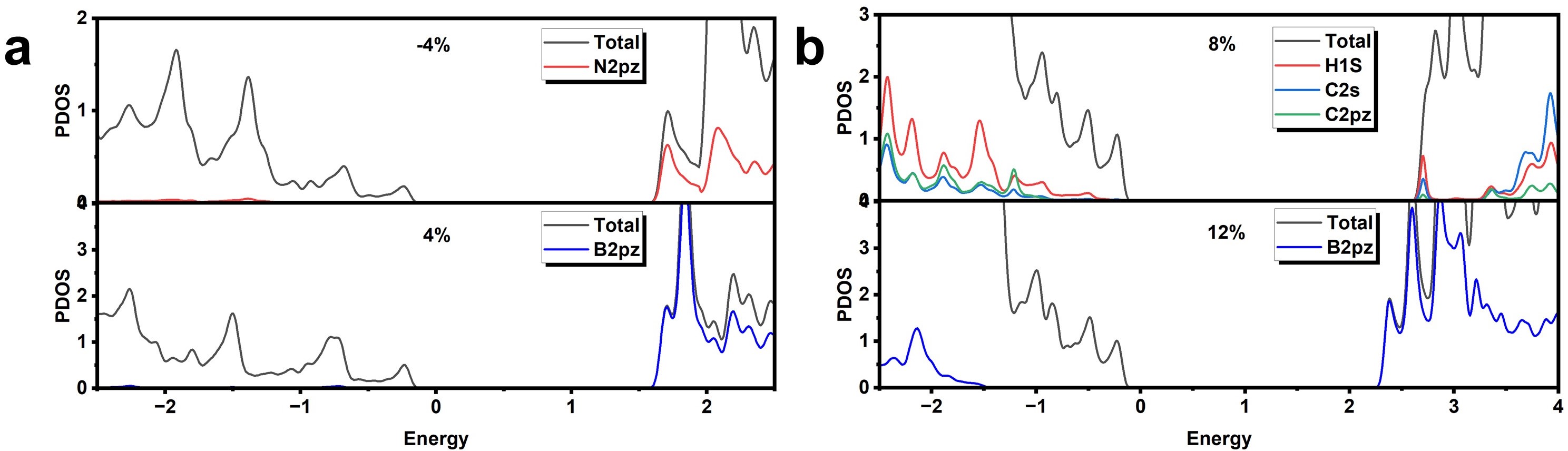


**Figure S5. PDOS diagrams of the a. h-BN/H-diamond system at -4% and 4% biaxial strain and b. h-BN/OH-diamond system at 8% and 12% biaxial strain.**

As shown in Fig. S5a, it can be concluded that the direct-indirect transition in the band gap of the h-BN/H-diamond system occurs due to the change in orbital hybridisation between the atoms. Here the CBM of the h-BN/H-diamond (under -4% strain) system is contributed by the N2pz orbitals, while the CBM of the h-BN/H-diamond (at 4% strain) system is contributed by the B2pz orbitals. Similarly, the CBM of the h-BN/OH-diamond (under 8% strain) system is contributed by the H1S, C2, and C2pz orbitals, whereas the CBM of the h-BN/OH-diamond (at 12% strain) system is contributed by the B2pz orbitals (shown in Fig. S5b).

**
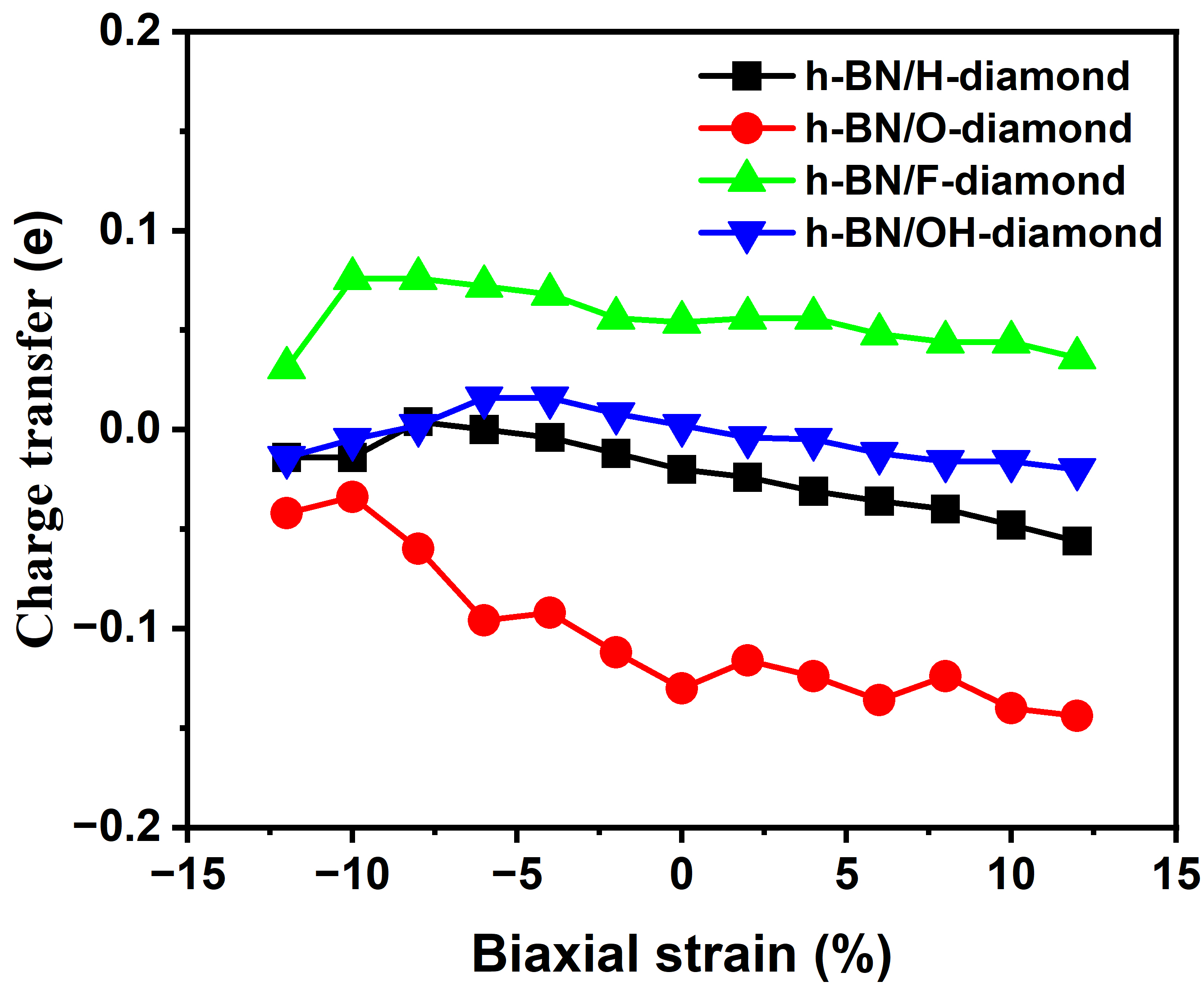
**

**Figure S6. Transferred charge of the h-BN/(H,O,F,OH)-diamond system at strain of -12%~12%.**

Taking the h-BN/F-diamond system as an example, the charge transfer decreases with increasing strain, leading to a weakening of the interaction between h-BN and F-diamond causing an increase in the band gap of the system. And the increase of band gap will spontaneously stimulate the upward movement of the bottom of the conduction band and the top of the valence band (e.g., Fig. S4), which induces the change of band offset, such as the decrease of valence band offset.
